# 2206 Chicago Kids Advisory Board: A novel approach to engaging adolescent students in pediatric clinical research

**DOI:** 10.1017/cts.2018.239

**Published:** 2018-11-21

**Authors:** Ferdynand Hebal, Susanna McColley

**Affiliations:** University of Chicago

## Abstract

OBJECTIVES/SPECIFIC AIMS: Stakeholder engagement has been proposed to help realign clinical and translational research with the needs of clinicians, patients, and policymakers. Increasingly, funders and researchers seek to partner with stakeholders to inform study design, execution and dissemination of results. Kids and families Impacting Disease through Science (KIDS) is a program of the American Academy of Pediatrics that seeks to engage youth in clinical research. United States KIDS programs participate in International Children’s Advisory Network activities. The Chicago KIDS Advisory Board program at Walter Payton College Preparatory School, a Chicago Public School, was initiated in 2015 to foster and develop interest in careers in science, research and healthcare and provide youth perspectives to academic and industry researchers on the design and development of pediatric research studies. This project engaged youth advisors in creation and evaluation of a video explaining clinical research and informed consent for Ann & Robert H. Lurie Children’s Hospital, a clinical partner of the Northwestern University Clinical and Translational Sciences Institute. METHODS/STUDY POPULATION: The Payton program advisory board sessions are 1.5hr interactive seminars held on 1–2 school days each month. During the 2016–2017 school year, students participated in 3 stakeholder sessions, led by Lurie Children’s hospital researchers, to advise development of a script, storyboards, and ultimately an animated video that informs children and families about participation in clinical research to aid in the decision-making process. Qualitative research methods were used to examine attitudes towards clinical research and assess the video on content objectives, clarity of concept, and appropriateness for a pediatric audience. Following production, students from the 2017–2018 advisory board viewed the final video and presurvey and postsurvey were administered to assess the effect of video on the comprehension of 8 key concepts of informed consent on a 5-point Likert scale. The Wilcoxon signed-rank test was used to compare median pretest and post-test ranks. Results of this analysis were reviewed in seminar and students provided written contribution to this abstract. RESULTS/ANTICIPATED RESULTS: In total, 11 Walter Payton high school students participated in video development and 27, who were naïve to development, participated in the pre and post evaluation sessions. Students ranged from Freshman to Seniors and reflected the diverse ethnic and racial background of Chicago. A positive change from pre to post-test survey was observed in all questions presented assessing comprehension of key concepts of informed consent. The median post-test ranks were statistically significantly higher than the median pre-test ranks for all questions (*p*<0.01 in all). DISCUSSION/SIGNIFICANCE OF IMPACT: Chicago KIDS youth advisors were engaged in all aspects of the design of the research tool and gained experience in stakeholder contribution from study design to evaluation and publication. The students will next be involved in the design of a prospective randomized study to test the efficacy of the video compared with standard recruitment and consent practices. Given the difficulty of recruiting youth for clinical trials, development of effective engagement practices in is critically important. Our findings demonstrate the feasibility of utilizing youth advisors in a public school based setting.

